# The impact of telephone follow up on adverse events for Aboriginal people with chronic disease in new South Wales, Australia: a retrospective cohort study

**DOI:** 10.1186/s12939-018-0776-2

**Published:** 2018-05-18

**Authors:** Amanda Jayakody, Erin Passmore, Christopher Oldmeadow, Jamie Bryant, Mariko Carey, Eunice Simons, Aaron Cashmore, Louise Maher, Kiel Hennessey, Jacinta Bunfield, Maurice Terare, Andrew Milat, Rob Sanson-Fisher

**Affiliations:** 1 Health Behaviour Research Collaborative, School of Medicine and Public Health, Faculty of Health and Medicine, University of Newcastle, Callaghan, NSW 2308 Australia; 2 Priority Research Centre for Health Behaviour, University of Newcastle, Callaghan, 2308 NSW Australia; 3 Hunter Medical Research Institute, New Lambton Heights, 2305 NSW Australia; 4 Evidence and Evaluation, Centre for Epidemiology and Evidence, NSW Ministry of Health LMB 961, North Sydney, Sydney, NSW 2059 Australia; 5 School of Medicine and Public Health, University of Newcastle, Callaghan, NSW 2308 Australia; 6 CREDITSS—Clinical Research Design, Information Technology and Statistical Support Unit, Hunter Medical Research Institute, HMRI Building, New Lambton Heights, 2305 NSW Australia; 7 NSW Agency for Clinical Innovation, Level 4, Sage Building, 67 Albert Ave, Chatswood, Sydney, NSW 2067 Australia; 8 School of Public Health and Community Medicine, University of NSW, Sydney, 2033 Australia; 9 Centre for Aboriginal Health, NSW Ministry of Health LMB 961, North Sydney, Sydney, NSW 2059 Australia; 10 Sydney Medical School, University of Sydney, Edward Ford Building A27, The University of Sydney, Sydney, NSW 2006 Australia

**Keywords:** Unplanned readmission, Telephone follow up, Aboriginal health, Health services research

## Abstract

**Background:**

Chronic diseases are more prevalent and occur at a much younger age in Aboriginal people in Australia compared with non-Aboriginal people. Aboriginal people also have higher rates of unplanned hospital readmissions and emergency department presentations. There is a paucity of research on the effectiveness of follow up programs after discharge from hospital in Aboriginal populations. This study aimed to assess the impact of a telephone follow up program, 48 Hour Follow Up, on rates of unplanned hospital readmissions, unplanned emergency department presentations and mortality within 28 days of discharge among Aboriginal people with chronic disease.

**Methods:**

A retrospective cohort of eligible Aboriginal people with chronic diseases was obtained through linkage of routinely-collected health datasets for the period May 2009 to December 2014. The primary outcome was unplanned hospital readmissions within 28 days of separation from any acute New South Wales public hospital. Secondary outcomes were mortality, unplanned emergency department presentations, and at least one adverse event (unplanned hospital readmission, unplanned emergency department presentation or mortality) within 28 days of separation. Logistic regression models were used to assess outcomes among Aboriginal patients who received 48 Hour Follow Up compared with eligible Aboriginal patients who did not receive 48 Hour Follow Up.

**Results:**

The final study cohort included 18,659 patients with 49,721 separations, of which 8469 separations (17.0, 95% confidence interval (CI): 16.7–17.4) were recorded as having received 48 Hour Follow Up. After adjusting for potential confounders, there were no significant differences in rates of unplanned readmission or mortality within 28 days between people who received or did not receive 48 Hour Follow Up. Conversely, the odds of an unplanned emergency department presentation (Odds ratio (OR) = 0.92; 95% CI: 0.85, 0.99; *P* = 0.0312) and at least one adverse event (OR = 0.91; 95% CI: 0.85,0.98; *P* = 0.0136) within 28 days were significantly lower for separations where the patient received 48 Hour Follow Up compared with those that did not receive follow up.

**Conclusions:**

Receipt of 48 Hour Follow Up was associated with both a reduction in emergency department presentations and at least one  adverse event within 28 days of discharge, suggesting there may be merit in providing post-discharge telephone follow up to Aboriginal people with chronic disease.

**Electronic supplementary material:**

The online version of this article (10.1186/s12939-018-0776-2) contains supplementary material, which is available to authorized users.

## Background

Aboriginal and Torres Strait Islander people in Australia (hereafter 'Aboriginal people') experience considerably poorer health outcomes compared with non-Aboriginal people, and also compared with other Indigenous people in New Zealand, Canada and the United States [1]. In Australia, Aboriginal men, on average, live 10.6 years less than non-Aboriginal men, while Aboriginal women, on average, live 9.5 years less than non-Aboriginal women [2] .This difference is largely accounted for by chronic diseases such as cardiovascular diseases, type 2 diabetes, chronic respiratory diseases and renal diseases, which are more prevalent among Aboriginal people and occur at a much younger age [3, 4].

Aboriginal people also have higher rates of unplanned hospital readmissions and emergency department (ED) presentations. In New South Wales (NSW), Australia, Aboriginal people are 1.3 times more likely to have an unplanned hospital readmission within 28 days of discharge from hospital, and 1.3 times more likely to re-present to the ED within 48 h compared with non-Aboriginal people [5]. These higher hospital utilisation rates may indicate the health needs of Aboriginal people are not being met during their hospital stay and post-discharge.

Telephone follow up (TFU) is a strategy that has been frequently used to support patients after discharge from hospital. TFU involves a hospital or community health professional telephoning a discharged patient at home to provide ongoing education and management of symptoms with the aim of reducing problems post-discharge [6]. Systematic reviews have highlighted that TFU is a common part of successful multi-component interventions in reducing readmissions in general medical and surgical patients [7, 8]. A systematic review by Jayakody and colleagues examined 10 interventions utilising TFU in combination with other strategies amongst patients with chronic disease [9]. Of the 10 studies identified, five were found to be effective in reducing unplanned readmission within 30 days of discharge. Interventions that were effective included three studies which provided TFU in addition to pre-discharge support; and two studies which provided TFU with both pre- and post-discharge support such as education, discharge planning, and home visits [9]. Despite a developing evidence base for the effectiveness of TFU in reducing unplanned hospital readmissions, no studies have examined the effectiveness of TFU in Aboriginal populations, either in Australia or elsewhere.

The 48 Hour Follow Up program is a NSW Health state government initiative to improve coordination and management of care for Aboriginal people with chronic diseases. The program aims to reduce unplanned hospital readmissions and improve health outcomes for Aboriginal people with a chronic disease. The program involves identifying, from hospital records, Aboriginal people meeting the following eligibility criteria: 1) Aboriginal and/or Torres Strait Islander person; 2) aged 15 years and older; 3) admitted to an acute care facility; and 4) has a chronic disease. An attempted telephone call is made to the identified eligible patient within two working days of discharge from an acute care facility. The phone call is ideally conducted by an Aboriginal health professional but in some cases a non-Aboriginal staff member may make the call. The call covers, at a minimum: access to medications; whether the patient has referrals and follow up appointments in place; and general wellbeing. The caller seeks to identify and resolve any issues with the patient’s post-discharge care, and to ensure appropriate links to general practitioners, Aboriginal Community Controlled Health Services, specialists and other services that are able to assist the patient with receiving appropriate post-discharge care. The program commenced in 2009 and is currently delivered in all 15 local health districts (LHD) (government corporations responsible for managing public hospitals and providing health services to defined geographical areas of the state) in NSW.

Due to the high rates of chronic disease and unplanned hospital readmission amongst Aboriginal people, it is critical to develop evidence about what strategies are effective in reducing unplanned readmission within 28 days of discharge. This study addresses the paucity of research evidence in this field for Aboriginal populations by providing state-wide data on the impact of a telephone follow up service for Aboriginal people with chronic disease. It provides novel data to assist policy makers in determining the impact of the 48 Hour Follow Up program and determining its future directions. Furthermore, this study provides a unique evaluation of a system-level intervention which is rarely published in the literature [10, 11]. Specifically, the study aims to assess the impact of the 48 Hour Follow Up program on rates of unplanned hospital readmissions within 28 days of discharge among Aboriginal people with chronic disease in NSW. As a secondary aim the impact of the program on unplanned ED presentations, mortality and at least one adverse event (unplanned hospital readmission, unplanned ED presentation or  mortality) within 28 days of discharge were also examined.

## Methods

### Study design

A retrospective cohort was obtained through linkage of routinely-collected health datasets.

### Data sources

Data were obtained from the 48 Hour Follow Up Program Register, a public health register established under the *NSW Public Health Act 2010* [12], comprising linked data from the following five sources:

1. *48 Hour Follow Up Program Dataset* comprises records from each participating LHD for all patients identified by the LHD as eligible to receive 48 Hour Follow Up. Data includes the dates of admission and separation from hospital, unique patient identifiers and whether or not the patient received 48 Hour Follow Up;
2. *NSW Admitted Patient Data Collection (APDC)* comprises records of all separations in NSW private and public hospitals, including discharges, transfers and deaths;
3. *NSW Register of Births, Deaths and Marriages (RBDM)* provides fact of death for all deaths registered in NSW;
4. *NSW Emergency Department Data Collection (EDDC*) comprises records of all presentations to EDs in NSW public hospitals;
5. *NSW Chronic Disease Management Program (CDMP) Minimum Dataset* comprises records from the NSW Health Chronic Disease Management Program, which provides care coordination and self-management support to people with chronic disease. The dataset also holds information on participation in Healthways, a telephone health coaching service offered to a subset of CDMP participants (this service was offered in a number of LHDs to patients who had one unplanned hospital admission in the previous 12 months relating to one of the five chronic conditions that were part of the CDMP (i.e. diabetes, hypertension, coronary artery disease, heart failure, chronic obstructive pulmonary disease)).

### Study population

#### Eligibility criteria

The study sample included all patients who met the eligibility criteria of the 48 Hour Follow Up Program in the period May 2009 to December 2014. Eligible patients were: 1) Aboriginal and/or Torres Strait Islander people; 2) aged 15 years and older at the time of admission; 3) admitted to an acute facility in a NSW public hospital; 4) discharged from hospital to the community; and 5) had one or more of the following ICD-10-defined chronic diseases as a principal or additional diagnosis: cardiovascular disease, diabetes, respiratory disease and renal diseases (Additional file 1). Eligible patients for this data analysis included all those identified in the 48 Hour Follow Up Program Dataset (indicating LHD staff assessed the patient as eligible for 48 Hour Follow Up), plus patients identified through the APDC as meeting the eligibility criteria for 48 Hour Follow Up.

#### Exclusion criteria

Patients were excluded if they were readmitted to hospital within 48 h of discharge, as the LHD may not have been able to follow up the patient prior to the readmission. Duplicate records were also excluded.

### Data linkage

The data sources were linked by the Centre for Health Record Linkage using probabilistic record linkage methods [13]. Following linkage, disease-related, sociodemographic and admission variables (see *Explanatory variables* below) were derived from the APDC dataset. Any missing data were populated from the 48 Hour Follow Up Program dataset. The exception to this was Aboriginality; patients were considered an Aboriginal person if they were listed as being an Aboriginal and/or Torres Strait Islander person on any APDC record, or if they were identified in the 48 Hour Follow Up Program dataset (May 2009 to December 2014). This method was selected based on the advice of the study’s advisory committee, which had Aboriginal representation. This was considered the most accurate method available for retrieving Aboriginal status. The APDC is estimated to correctly report Aboriginal status at a level of 90.7% (95% CI 84.6–94.2) [14]. The inclusion and exclusion criteria were then applied to give the final dataset for analysis. All data were de-identified.

### Analysis variables

#### Primary and secondary outcomes

The primary outcome was unplanned hospital readmissions within 28 days of separation from any acute facility in a NSW public hospital. ‘Unplanned’ refers to emergency admissions where admission is required within 24 h of diagnosis. Readmission refers to an admission with an admission date within 28 days of the discharge date for any purpose other than mental health, chemotherapy or dialysis. Transfers from other facilities were not considered as readmissions, and excluded from analysis. Secondary outcomes were mortality within 28 days of separation; unplanned ED presentation within 28 days of separation (‘unplanned’ refers to either an ‘emergency presentation’ or an ‘unplanned return visit for continuing condition’ as reported in the EDDC); and an “adverse event” defined as mortality, unplanned ED presentation or unplanned hospital readmission within 28 days of separation.

#### Explanatory variables

The main explanatory variable of interest was receipt of 48 Hour Follow Up. Eligible individuals were classified as either having: 1) received 48 Hour Follow Up; or 2) not received 48 Hour Follow Up. The 48 Hour Follow Up Program Dataset reports whether the patient was followed up within two working days of discharge or outside two working days. Sensitivity analyses, conducted to examine any significant differences between the two timeframes of follow up, found results were broadly similar (results are provided in Additional file 2). Therefore, for the purpose of this analysis patients followed up both within or outside two working days were classified as having received 48 Hour Follow Up.

Based on discussions with content experts and a review of the literature the following variables were considered as potential confounders:

- Models of care: Although all LHDs implement the 48 Hour Follow Up phone call, the model of program delivery varies. There are four primary models: 1) a *centralised* model, where all 48 Hour Follow Up phone calls for the LHD are conducted from a central call centre (five LHDs implement this model); 2) a *shared care* model, where there is close integration between hospital and community-based services, and the most appropriate staff member conducts the phone call (three LHDs); 3) a *localised* model, where an Aboriginal Health Worker at each inpatient facility conducts the phone call, and sometimes also a home visit (six LHDs); and 4) an *Aboriginal Medical Service (AMS)-contracted* model, where the local AMS conducts calls to patients currently case-managed by the AMS, and the LHD conducts calls to non-AMS patients (one LHD).
- Enrolment in a CDMP or a Healthways program at the date of separation.
- Disease-related variables: Charlson Comorbidity Index [15], and the number of additional diagnoses (less than 2; 2 or more).
- Sociodemographic variables: Patient’s gender, age, marital status, and Index of Relative Socio-economic Disadvantage (IRSD) quintile. The IRSD is a general socio-economic index that summarises a range of information about the economic and social conditions of people and households within a geographic area [16].
- Admission variables: The following variables were collected for each hospital admission: year of admission, length of stay, and number of previous admissions within the study period.

### Statistical analysis

Logistic regression models were used to assess rates of unplanned hospital readmissions, mortality, unplanned ED presentations and adverse events within 28 days of discharge among Aboriginal patients who received 48 Hour Follow Up compared with eligible Aboriginal patients who did not receive 48 Hour Follow Up. Potentially confounding variables (models of care, enrolment in a CDMP or Healthways program, Charlson Comorbidity Index, number of additional diagnoses, sociodemographic factors, year of admission, length of stay and number of previous admissions) were identified by comparing the characteristics of patients who received 48 Hour Follow Up compared with eligible patients who did not receive 48 Hour Follow Up using logistic regression models. Variables which were associated at a 5% significance level with both receipt of 48 Hour Follow Up and the adverse event were included in the logistic regression models. Clustering from repeated admissions for the same patient was accounted for by using generalised estimating equations with an exchangeable correlation structure, and robust Hubert-White standard errors were used. A detailed description of this analysis is available online as part of a wider evaluation report. [17] All analyses were done using SAS 9.4 software [18].

The study was approved by the University of Newcastle Human Research Ethics Committee (H-2013-0381) and the NSW Aboriginal Health and Medical Research Council Ethics Committee (967/13).

## Results

In the linked dataset there were 407,729 hospital separations for Aboriginal patients aged 15 years or older who attended a public hospital in NSW between May 2009 and December 2014. Of these, 350,954 separations which did not meet the 48 Hour Follow Up program criteria were excluded. An additional 7054 records were removed due to being duplicate records or because the separation was followed by a readmission within 48 h. The final study cohort included 18,659 Aboriginal patients, with 49,721 separations. The mean number of separations per patient was 2.6 (Standard deviation (SD) = 4.5).

Of the 49,721 eligible separations, 8469 (17.0%, 95% CI 16.7–17.4) were recorded as having received 48 Hour Follow Up. Among patients who received 48 Hour Follow Up, 73.6% (*n* = 6230) were followed up within two working days of discharge from hospital, and the remaining 26.4% (*n* = 2239) were followed up outside two working days. Table 1 presents the characteristics associated with receiving 48 Hour Follow Up, adjusted for model of care and year. Relative to the least socially disadvantaged quintile (1st quintile), all increasing quintiles of social disadvantage had higher odds of follow up (2nd quintile OR = 1.44; 95% CI = 1.30, 1.60; 3rd quintile OR = 1.37; 95% CI = 1.24, 1.52; 4th quintile OR = 1.09; 95% CI = 0.98, 1.21; 5th quintile OR = 1.38; 95% CI = 1.24, 1.54). Hospital stays of longer than one day had higher odds of being followed up compared with stays of one day or less (OR = 1.28; 95% CI = 1.21, 1.35). Patients with two or more diagnoses had lower odds of being followed up compared with patients with less than two diagnoses (OR = 0.80; 95% CI = 0.75, 0.86). Similarly, patients with a higher Charlson Comorbidity Index had lower odds of follow up (OR = 0.72; 95% CI = 0.70, 0.75). There was no significant association between gender, age, marital status, participation in CDMP and Healthways, and the number of previous admissions with receiving 48 Hour Follow Up.

**Table 1:** Characteristics associated with receiving 48 Hour Follow Up among Aboriginal patients, adjusted for model of care and year (*N* = 49,721)*

		N (%)			
Variable	Category	Not followed up (n = 41,252)	Followed up (n = 8469)	OR (95% CI)	*P*-value
Gender	Male	18,765 (83%)	3818 (17%)	ref	0.9040
	Female	22,319 (83%)	4591 (17%)	1.00 (0.94,1.07)	
Marital status	Married/de facto	15,448 (84%)	2874 (16%)	ref	0.1269
	Single	13,710 (84%)	2619 (16%)	1.01 (0.93,1.09)	
	Widowed	5185 (86%)	877 (14%)	1.04 (0.92,1.17)	
	Divorced/separated	5672 (85%)	1014 (15%)	1.03 (0.93,1.14)	
	Not known	441 (92%)	37 (7.7%)	0.63 (0.44,0.90)	
IRSD quintile	1st quintile - least disadvantaged	7098 (87%)	1087 (13%)	ref	<.0001
	2nd quintile	7631 (82%)	1642 (18%)	1.44 (1.30,1.60)	
	3rd quintile	9039 (83%)	1809 (17%)	1.37 (1.24,1.52)	
	4th quintile	8016 (86%)	1357 (14%)	1.09 (0.98,1.21)	
	5th quintile - most disadvantaged	8671 (85%)	1484 (15%)	1.38 (1.24,1.54)	
Participation in CDMP	Did not participate	40,206 (83%)	8160 (17%)	ref	0.1445
	Participated	1046 (77%)	309 (23%)	1.16 (0.95,1.40)	
Participation in Healthways	Did not participate	41,230 (83%)	8456 (17%)	ref	0.1270
	Participated	22 (63%)	13 (37%)	1.91 (0.83,4.41)	
Length of stay	1 day or less	15,652 (85%)	2729 (15%)	ref	<.0001
	More than 1 day	24,835 (84%)	4692 (16%)	1.28 (1.21,1.35)	
No. of previous admissions	None	15,269 (82%)	3390 (18%)	ref	0.3259
	1 or more	25,983 (84%)	5079 (16%)	0.97 (0.92,1.03)	
No. of additional diagnoses	Less than 2	11,118 (76%)	3526 (24%)	ref	<.0001
	2 or more	30,134 (86%)	4943 (14%)	0.80 (0.75,0.86)	
Age	mean (SD)	55 (16)	53 (18)	1.00 (1.00,1.00)**	0.9970
Charlson Comorbidity Index	mean (SD)	2 (1)	1 (2)	0.72 (0.70,0.75)***	<.0001

*Frequencies are calculated using all eligible hospital separations (N = 49,721). Odds ratios are calculated for hospital separations with complete patient characteristic data (*N* = 47,803)

**Odds ratio is the increase in odds for each additional year of age. ***Odds ratio is the increase in odds for each one unit increase on Charlson Comorbidity Index

Data source: 48 Hour Follow Up Program Register. Study period: May 2009 to December 2014 [17]

### Adverse events following hospital separation

Table 2 presents rates of adverse events within 28 days of discharge from hospital. Compared with eligible patients who did not receive 48 Hour Follow Up, patients who received 48 Hour Follow Up had lower rates of unplanned hospital readmissions, unplanned ED presentations, mortality and adverse events (unplanned readmission, unplanned ED presentation or mortality) within 28 days of discharge from hospital (Table 2).

**Table 2:** Hospital separations of Aboriginal patients that resulted in an adverse event within 28 days of discharge, by whether the patient received 48 Hour Follow Up (*n* = 49,721)

Variable	Number of events among separations that did not receive 48 Hour Follow Up *N* (%) (*n* = 41,252)	Number of events among separations that received 48 Hour Follow Up *N* (%) (*n* = 8469)	Total number with event (% of total sample)
Unplanned hospital readmission within 28 days	3119 (7.6%)	455 (5.4%)	3574 (7.2%; 95% CI 7.0, 7.4)
Mortality within 28 days	460 (1.1%)	75 (0.9%)	535 (1.1%; 95% CI 0.98, 1.2)
Unplanned ED presentation within 28 days	9535 (23%)	1745 (21%)	11,280 (22.7, 95% CI 22.3, 23.0)
At least 1 adverse event	10,136 (25%)	1810 (21%)	11,946 (24%; 95% CI 23.6, 24.4)

Data source: 48 Hour Follow Up Program Register. Study period: May 2009 to December 2014 [17]

### Rates of adverse events by receipt of 48 Hour Follow Up

Results of multivariable logistic regression modelling of adverse events associated with receipt of 48 Hour Follow Up, which are adjusted for all variables given in the table, are shown in Table 3. After adjusting for potential confounders, there was no statistically significant association between receipt of 48 Hour Follow Up and unplanned readmission or mortality within 28 days of discharge.

**Table 3:** Association between receipt of 48 Hour Follow Up and adverse events among Aboriginal patients: Logistic GEE models adjusting for variables associated with both receipt of 48 Hour Follow Up and the adverse event (n = 49,721)*

		Unplanned readmission		Mortality		ED presentation		At least 1 adverse event	
Variable	Category	OR (95% CI)	*P*-value	OR (95% CI)	*P*-value	OR (95% CI)	*P*-value	OR (95% CI)	*P*-value
Follow up	Not followed up	reference	0.1352	reference	0.4760	reference	0.0312	reference	0.0136
	Followed up	0.84 (0.66,1.06)		0.91 (0.69,1.19)		0.92 (0.85,0.99)		0.91 (0.85,0.98)	
Care type	Centralised					reference	<.0001	reference	<.0001
	Shared care					1.00 (0.91,1.10)		1.02 (0.93,1.11)	
	Localised					0.77 (0.72,0.83)		0.81 (0.76,0.87)	
	AMS-contracted					1.01 (0.76,1.33)		0.94 (0.72,1.24)	
Year	2009			reference	0.0003	reference	<.0001	reference	<.0001
	2010			1.19 (0.81,1.73)		1.10 (0.99,1.23)		1.06 (0.95,1.18)	
	2011			1.40 (0.96,2.05)		1.24 (1.11,1.38)		1.20 (1.08,1.33)	
	2012			1.50 (1.06,2.12)		1.29 (1.15,1.43)		1.22 (1.10,1.35)	
	2013			1.50 (1.08,2.08)		1.32 (1.19,1.46)		1.24 (1.12,1.37)	
	2014			0.75 (0.50,1.12)		1.21 (1.07,1.36)		1.12 (1.00,1.26)	
IRSD quintile	1st quintile - least disadvantaged		.		.	reference	<.0001	reference	0.0005
	2nd quintile					0.91 (0.82,1.00)		0.92 (0.83,1.01)	
	3rd quintile					0.99 (0.89,1.09)		0.99 (0.91,1.09)	
	4th quintile					0.82 (0.74,0.90)		0.85 (0.77,0.94)	
	5th quintile - most disadvantaged					0.80 (0.72,0.89)		0.85 (0.77,0.94)	
Length of stay	1 day or less		.	reference	<.0001	reference	0.0007	reference	<.0001
	More than 1 day			1.93 (1.53,2.42)		1.11 (1.04,1.17)		1.13 (1.06,1.19)	
No. of additional diagnoses	Less than 2	reference	0.2646	reference	0.1804	reference	0.0187	reference	0.0870
	2 or more	1.13 (0.91,1.40)		1.24 0.91,1.68)		1.09 (1.01,1.17)		1.06 (0.99,1.14)	
Charlson Comorbidity Index		1.11 (1.06,1.17)	<.0001	1.46 (1.40,1.53)	<.0001	1.08 (1.06,1.11)	<.0001	1.10 (1.08,1.13)	<.0001

*Odds ratios are from the logistic regression GEE model and are adjusted for all variables given in the table

Data source: 48 Hour Follow Up Program Register. Study period: May 2009 to December 2014 [17]

After adjusting for potential confounders, the odds of an unplanned ED presentation within 28 days were significantly lower for separations where the patient received 48 Hour Follow Up compared with those that did not receive 48 Hour Follow Up (OR = 0.92; 95% CI: 0.85, 0.99; *P* = 0.0312). The adjusted odds of at least one adverse event for those that received 48 Hour Follow Up was also significantly lower (OR = 0.91; 95% CI: 0.85, 0.98; *P* = 0.0136) compared with separations that did not receive 48 Hour Follow Up.

## Discussion

To the best of our knowledge, this is the first study to examine the effectiveness of TFU for recently discharged Aboriginal people with chronic disease. While there was no evidence of an effect of the 48 Hour Follow Up program on unplanned readmissions or mortality within 28 days of discharge, receipt of 48 Hour Follow Up was significantly associated with both  fewer unplanned ED presentations and at least one adverse event within 28 days of discharge.

There are a number of potential reasons for the lack of a significant reduction in unplanned hospital readmissions. Firstly, the target population for 48 Hour Follow Up (i.e. all Aboriginal people aged 15 years and older, with a specified chronic disease and who have been discharged from hospital) is broad, and there is no state-wide protocol for prioritizing the order in which patients receive 48 Hour Follow Up. Melton and colleagues conducted a randomised controlled trial among patients with gastrointestinal, heart, and lower respiratory diagnoses [19]. The intervention group received TFU within 24 h of discharge, and calls were prioritized so that patients with the greatest likelihood of readmission due to poorer health status were contacted first. A control group received TFU three days after discharge, and calls were not made in any health risk order. The prioritized treatment group had significantly fewer 30 day intent-to-treat readmissions (5.7% vs 7.3%; *p* < .05) compared with the non-prioritized control group [19]. This suggests the effectiveness of 48 Hour Follow Up may be enhanced by prioritizing ‘high risk’ patients for earlier follow up. However, there remain gaps in the evidence of what makes an effective TFU program. Mistiaen and Poot in their systematic review of TFU stress the need for further research to establish the ideal person to make the follow up call, the frequency and timing of calls, the content of the calls, and to identify the potential patient, health system and country differences in TFU interventions [6].

A second potential reason for the lack of a significant reduction in readmissions may be due to 48 Hour Follow Up being a standalone intervention. Although some LHDs have expanded the program to have additional components (e.g. in the localised model, some patients receive home visits), the centralised model delivers TFU as a standalone strategy. Hansen and colleagues comment on the merit of “bridging interventions” which combine pre- and post-discharge care to act as a “bridge” between hospital-, home- and community-based health care. [8] Studies conducted with other population groups have demonstrated the effectiveness of multi-component programs incorporating TFU with other intervention strategies such as discharge planning, patient education, home visits and transition coaching. [7, 8] For example, a non-randomised trial by Sales and colleagues amongst cardiac patients used trained volunteers to provide pre-discharge patient education and medication instructions and post-discharge TFU [20]. Compared with standard care, the intervention group had lower rates of 30-day readmissions [20]. Jayakody and colleagues in their systematic review of interventions utilising TFU amongst patients with chronic disease found all 10 included studies combined TFU with other components [9]. Although they report that the studies did not uniformly demonstrate significant reductions in readmission rates, the findings did suggest some merit in combining TFU with pre-discharge interventions such as discharge planning and patient education. Therefore standalone TFU interventions such as the 48 Hour Follow Up program may be strengthened by being combined with other interventions.

The association of 48 Hour Follow Up with both reductions in unplanned ED presentations and at least one adverse event is encouraging. Our results are similar to an intervention conducted in the United States by Dudas and colleagues who randomly assigned general medicine patients to receive a telephone call from a pharmacist within two days of discharge [21]. The study resulted in a significant reduction in unplanned ED presentations but not readmissions within 30 days. Although the patient group and person making the call were different to the 48 Hour Follow Up program, the call timing and content were similar. TFU calls may improve patients’ ability to self-manage their health issues and/or connect with community-based health services such as their general practitioner, rather than presenting to the ED. A key strength of TFU is its relative ease of implementation: it is less labour intensive than interventions such as home visits, is low cost, and is scalable to reach large populations [6, 22]. Although 48 Hour Follow Up did not significantly reduce unplanned hospital readmissions, the findings related to reduced unplanned ED presentations and adverse events suggest the program has some health benefits for patients. Future research may seek to identify which program characteristics (e.g. inclusion of home visits, whether the person conducting the 48 Hour Follow Up call is an Aboriginal person) influence the impact of 48 Hour Follow Up. In addition, the potential economic and health system benefits of reduced hospital utilization are worthy of further study [21].

Our study found that patients with more comorbidities (i.e. a higher Charlson Comorbidity Index and those with two or more chronic diseases) had lower odds of receiving 48 Hour Follow Up. This was an unexpected finding as there is no prioritisation of patients for 48 Hour Follow Up, and therefore we would expect there to be no association between comorbidity and receipt of follow up. A similar “treatment-risk paradox” [23] was observed by Wong and colleagues, who found that among patients presenting to Canadian EDs with chest pain, those with more comorbidities were less likely to receive physician follow-up after discharge [24]. One possible explanation is that 48 Hour Follow Up staff may refer patients with comorbidities directly to other chronic disease management programs more tailored to supporting patients with complex needs, without conducting the 48 Hour Follow Up call; however 48 Hour Follow Up program staff interviewed in our process evaluation did not indicate that this was the case [17]. Another possible explanation is that patients with comorbidities may be less likely to answer or accept the 48 Hour Follow Up call, for example because they are already linked with other community-based health services and do not feel a need for additional support. Regardless of the reason why patients with comorbidities were less likely to receive 48 Hour Follow Up, this finding highlights the importance of prioritising high-risk patients to receive 48 Hour Follow Up, and more broadly the importance of integration of services to ensure patients with comorbidities do not fall through the net of service delivery.

### Limitations

This evaluation had a number of limitations. Firstly, an experimental design such as a randomised controlled trial would have provided the most robust information about effectiveness. However, randomized designs are not always feasible for population-level interventions [25]. In this case it was not possible given the program has been implemented state-wide for several years, aims to reach the entire population of Aboriginal people with chronic disease, and has variability in implementation across LHDs. A non-randomised cohort design was considered the most feasible approach to balance the tension between scientific rigour and the practicalities of evaluating an established state-wide government program. Secondly, the study relied on routinely-collected health data, rather than data collected for research purposes. Some limitations of routinely-collected data include the possibility of underreporting of Aboriginality in hospital data [14], and limited capacity to adjust for confounding variables. For example socio-economic status, a strongly confounding variable in this study, was measured based on patients’ postcode, and therefore may not have been an accurate measure of individuals’ socio-economic status. Thirdly, as the analysis explored one primary and three secondary outcomes, there is an elevated risk of declaring spuriously positive associations.

## Conclusions

The effectiveness of TFU in reducing adverse events has not previously been shown for Aboriginal people. Such findings help address the paucity of published research describing the effectiveness of policies and programs that target Aboriginal people. Our study found that the 48 Hour Follow Up program was not associated with reduced hospital readmissions or mortality within 28 days of hospital discharge among Aboriginal people with a chronic disease. However, receipt of 48 Hour Follow Up was associated with both a significant reduction in unplanned ED presentations and at least one  adverse event (hospital readmission, ED presentation or mortality) within 28 days of discharge, suggesting there may be some merit in providing post-discharge TFU to Aboriginal people with chronic disease.

## Additional Files

Additional file 1: ICD-10 codes used for 48 h follow up (Principle or an additional diagnosis). A list of all ICD-10 codes for chronic diseases meeting the eligibility cireria for the 48 Hour Follow Up program. (DOCX 22 kb)

Additional file 2: Sensitivity analysis results. Results of sensitivity analyses: factors associated with being followed up either within or outside 48 h; summary of the number of admissions that resulted in an adverse event by whether or not they received Follow Up; and Crude (Unadjusted) Models for “Not followed up” compared to “Followed up within 48 hours”. (DOCX 29 kb)

## Abbreviations

AMS: Aboriginal Medical Service
APDC: NSW Admitted Patient Data Collection
CDMP: NSW Chronic Disease Management Program
CI: Confidence interval
ED: Emergency department
EDDC: NSW Emergency Department Data Collection
IRSD: Index of Relative Socio-economic Disadvantage
LHD: Local health districts
NSW: New South Wales
OR: Odds ratio
RBDM: NSW Register of Births, Deaths and Marriages
SD: Standard deviation
TFU: Telephone follow up
