# Supplementary material for: The impact of telephone follow up on adverse events for Aboriginal people with chronic disease in new South Wales, Australia: a retrospective cohort study
Source: Int J Equity Health. 2018 May 18;17:60. doi: 10.1186/s12939-018-0776-2 (PMC5960116; doi:10.1186/s12939-018-0776-2)
Supplement: Supplementary file 1 — ICD-10 codes used for 48 h follow up (Principle or an additional diagnosis). A list of all ICD-10 codes for chronic diseases meeting the eligibility cireria for the 48 Hour Follow Up program. (DOCX 22 kb) [file 12939_2018_776_MOESM1_ESM.docx]

**Additional File 1: ICD-10 codes used for 48 hour follow up *(Principle or an additional diagnosis)***

| **ICD code description** | **Code** | **ICD code description** | **Code** |
| --- | --- | --- | --- |
| Asthma | J45 | Cerebrovascular disorders in dis cl/e | I68 |
| Status asthmaticus | J46 | Sequelae of cerebrovascular disease | I69 |
| Rheumatic mitral valve diseases | I05 | Emphysema | J43 |
| Rheumatic aortic valve diseases | I06 | Other chronic obstructive pulmonary disease | J44 |
| Rheumatic tricuspid valve diseases | I07 | Imparied glucose regulation | E09 |
| Multiple valve diseases | I08 | Type 1 diabetes mellitius | E10 |
| Other rheumatic heart diseases | I09 | Type 2 diabetes mellitius | E11 |
| Angina pectoris | I20 | Other specified diabetes mellitius | E13 |
| Acute myocardial infarction | I21 | Unspecified diabetes mellitius | E14 |
| Subsequent myocardial infarction | I22 | Acute nephritic syndrome | N00 |
| Certain current comp following acute MI | I23 | Rapidly progressive nephritic syndrome | N01 |
| Other acute ischaemic heart diseases | I24 | Recurrent and persistent haematuria | N02 |
| Chronic ischaemic heart disease | I25 | Chronic nephritic syndrome | N03 |
| Acute pericarditis | I30 | Nephrotic syndrome | N04 |
| Other diseases of pericardium | I31 | Unspecified nephritic syndrome | N05 |
| Pericarditis in dis classified elsewhere | I32 | Isolated proteinuria with specified morphological lesion | N06 |
| Acute and subacute endocarditis | I33 | Hereditary nephropathy, not elsewhere classified | N07 |
| Nonrheumatic mitral valve disorders | I34 | Glomerular disorders in diseases classified elsewhere | N08 |
| Nonrheumatic aortic valve disorders | I35 | Acute tubulo-interstitial nephritis | N10 |
| Nonrheumatic tricuspid valve disorders | I36 | Chronic tubulo-interstitial nephritis | N11 |
| Pulmonary valve disorders | I37 | Tubulo-interstitial nephritis not specified as acute or chronic | N12 |
| Endocarditis valve unspecified | I38 |  |  |
| Endocarditis heart valve disrd dis cl/e | I39 | Obstructive and reflux uropathy | N13 |
| Acute myocarditis | I40 | Drug- and heavy-metal-induced tubulo-interstitial and tubular conditions | N14 |
| Myocarditis in diseases class elsewhere | I41 |  |  |
| Cardiomyopathy | I42 | Other renal tubulo-interstitial diseases | N15 |
| Cardiomyopathy in diseases cl/e | I43 | Renal tubulo-interstitial disorders in diseases classified elsewhere | N16 |
| Atrioventricular & L bundle branch block | I44 |  |  |
| Other conduction disorders | I45 | Acute renal failure | N17 |
| Cardiac arrest | I46 | Chronic renal failure | N18 |
| Paroxysmal tachycardia | I47 | Unspecified renal failure | N19 |
| Atrial fibrillation and flutter | I48 | Calculus of kidney and ureter | N20 |
| Other cardiac arrhythmias | I49 | Calculus of lower urinary tract | N21 |
| Heart failure | I50 | Calculus of urinary tract in diseases classified elsewhere | N22 |
| Comp & ill-def description heart disease | I51 | Unspecified renal colic | N23 |
| Other heart disrd in dis class elsewhere | I52 | Disorders resulting from impaired renal tubular function | N25 |
| Subarachnoid haemorrhage | I60 | Unspecified contracted kidney | N26 |
| Intracerebral haemorrhage | I61 | Small kidney of unknown cause | N27 |
| Oth nontraumatic intracranial haem | I62 | Other disorders of kidney and ureter, not elsewhere classified | N28 |
| Cerebral infarction | I63 |  |  |
| Occlus precereb art no cereb infrct | I65 | Other disorders of kidney and ureter in diseases classified elsewhere | N29 |
| Occlus stenos cereb art no cereb infrct | I66 |  |  |
| Other cerebrovascular diseases | I67 |  |  |
