# Supplementary material for: The impact of telephone follow up on adverse events for Aboriginal people with chronic disease in new South Wales, Australia: a retrospective cohort study
Source: Int J Equity Health. 2018 May 18;17:60. doi: 10.1186/s12939-018-0776-2 (PMC5960116; doi:10.1186/s12939-018-0776-2)
Supplement: Supplementary file 2 — Sensitivity analysis results. Results of sensitivity analyses: factors associated with being followed up either within or outside 48 h; summary of the number of admissions that resulted in an adverse event by whether or not they received Follow Up; and Crude (Unadjusted) Models for “Not followed up” compared to “Followed up within 48 hours”. (DOCX 29 kb) [file 12939_2018_776_MOESM2_ESM.docx]

**Sensitivity analysis results**

*Table 1. Factors associated with being followed up either within or outside 48 hours*

|  | | **LHD eligible data (N = 10302)** | | | | **HIE eligible data (N = 47908)** | | | |
| --- | --- | --- | --- | --- | --- | --- | --- | --- | --- |
|  | | **N (%)** | | **GEE model** | | **N (%)** | | **GEE model** | |
| **Variable** | **Category** | **Not followed up (n=3384)** | **Followed up within 48 hours (n=5181)** | **OR (95% CI)** | **P-val** | **Not followed up (n=40487)** | **Followed up within or outside 48 (n=7421)** | **OR (95% CI)** | **P-val** |
| Model of care | Mixed LHD (FW) |  | 2 (100%) |  | <.0001 | 559 (93%) | 44 (7.3%) | 0.31 (0.21,0.44) | <.0001 |
|  | Other | 120 (65%) | 64 (35%) | 0.25 (0.17,0.38) |  | 2 (67%) | 1 (33%) |  |  |
|  | Centralised (SES HNE IS NBM SWS) | 1853 (35%) | 3388 (65%) | ref |  | 16324 (81%) | 3930 (19%) | ref |  |
|  | Integrated (MNC WS NSYD) | 795 (60%) | 527 (40%) | 0.46 (0.40,0.54) |  | 6392 (87%) | 972 (13%) | 0.55 (0.50,0.61) |  |
|  | Localised (SYD NNSW SNSW CC WNSW MUR) | 616 (34%) | 1200 (66%) | 1.01 (0.89,1.15) |  | 17210 (87%) | 2474 (13%) | 0.59 (0.55,0.63) |  |
| Year | 2009 | 98 (51%) | 93 (49%) | ref | <.0001 | 6674 (99%) | 93 (1.4%) | ref | <.0001 |
|  | 2010 | 433 (52%) | 402 (48%) | 0.65 (0.43,0.97) |  | 6163 (91%) | 575 (8.5%) | 5.96 (4.80,7.41) |  |
|  | 2011 | 604 (41%) | 863 (59%) | 1.05 (0.71,1.56) |  | 6215 (82%) | 1373 (18%) | 13.15 (10.61,16.30) |  |
|  | 2012 | 747 (38%) | 1233 (62%) | 1.22 (0.82,1.81) |  | 7652 (80%) | 1897 (20%) | 16.36 (13.23,20.24) |  |
|  | 2013 | 1279 (39%) | 1973 (61%) | 1.15 (0.78,1.70) |  | 9082 (77%) | 2685 (23%) | 20.53 (16.61,25.37) |  |
|  | 2014 | 223 (27%) | 617 (73%) | 1.99 (1.32,3.01) |  | 4701 (85%) | 798 (15%) | 13.22 (10.57,16.52) |  |
| Gender | Male | 1527 (39%) | 2407 (61%) | ref | 0.6947 | 18468 (85%) | 3384 (15%) | ref | 0.9040 |
|  | Female | 1857 (40%) | 2774 (60%) | 0.98 (0.89,1.08) |  | 22019 (85%) | 4037 (15%) | 1.00 (0.94,1.07) |  |
| Marital status | Married/defacto | 1230 (38%) | 2039 (62%) | ref | 0.0733 | 15448 (84%) | 2874 (16%) | ref | 0.1269 |
|  | Single | 1421 (43%) | 1854 (57%) | 0.85 (0.75,0.95) |  | 13710 (84%) | 2619 (16%) | 1.01 (0.93,1.09) |  |
|  | Widowed | 310 (35%) | 584 (65%) | 0.95 (0.78,1.14) |  | 5185 (86%) | 877 (14%) | 1.04 (0.92,1.17) |  |
|  | Divorced/separated | 397 (37%) | 673 (63%) | 1.00 (0.85,1.18) |  | 5672 (85%) | 1014 (15%) | 1.03 (0.93,1.14) |  |
|  | Not known | 26 (46%) | 31 (54%) | 0.79 (0.44,1.45) |  | 441 (92%) | 37 (7.7%) | 0.63 (0.44,0.90) |  |
| IRSD quintile | 1st quintile -least disadvantaged | 404 (33%) | 818 (67%) | ref | <.0001 | 7098 (87%) | 1087 (13%) | ref | <.0001 |
|  | 2nd quintile | 549 (30%) | 1279 (70%) | 1.14 (0.96,1.34) |  | 7631 (82%) | 1642 (18%) | 1.44 (1.30,1.60) |  |
|  | 3rd quintile | 1184 (50%) | 1199 (50%) | 0.67 (0.57,0.78) |  | 9039 (83%) | 1809 (17%) | 1.37 (1.24,1.52) |  |
|  | 4th quintile | 561 (38%) | 905 (62%) | 0.83 (0.70,0.99) |  | 8016 (86%) | 1357 (14%) | 1.09 (0.98,1.21) |  |
|  | 5th quintile - most disadvantaged | 642 (41%) | 932 (59%) | 0.92 (0.77,1.10) |  | 8671 (85%) | 1484 (15%) | 1.38 (1.24,1.54) |  |
| Participation in the CDMP | Did not participate | 3293 (40%) | 4959 (60%) | ref | 0.5342 | 39445 (85%) | 7125 (15%) | ref | 0.1445 |
|  | Participated | 91 (29%) | 222 (71%) | 1.10 (0.81,1.51) |  | 1042 (78%) | 296 (22%) | 1.16 (0.95,1.40) |  |
| Participation in the Healthways component | Did not participate | 3380 (40%) | 5170 (60%) | ref | 0.7886 | 40465 (85%) | 7408 (15%) | ref | 0.1270 |
|  | Participated | 4 (27%) | 11 (73%) | 1.11 (0.51,2.45) |  | 22 (63%) | 13 (37%) | 1.91 (0.83,4.41) |  |
| Length of stay | 1 day or less | 1511 (43%) | 1996 (57%) | ref | <.0001 | 15652 (85%) | 2729 (15%) | ref | <.0001 |
|  | More than 1 day | 1873 (37%) | 3185 (63%) | 1.26 (1.14,1.39) |  | 24835 (84%) | 4692 (16%) | 1.28 (1.21,1.35) |  |
| No of previous admissions | None | 2273 (40%) | 3370 (60%) | ref | 0.1548 | 14739 (84%) | 2872 (16%) | ref | 0.3259 |
|  | 1 or more | 1111 (38%) | 1811 (62%) | 1.08 (0.97,1.20) |  | 25748 (85%) | 4549 (15%) | 0.97 (0.92,1.03) |  |
| No of additional diagnoses | Less than 2 | 1091 (39%) | 1702 (61%) | ref | 0.0186 | 10353 (81%) | 2478 (19%) | ref | <.0001 |
|  | 2 or more | 2293 (40%) | 3479 (60%) | 0.88 (0.79,0.98) |  | 30134 (86%) | 4943 (14%) | 0.80 (0.75,0.86) |  |
| Age | mean (SD) | 50 (17) | 52 (18) | 1.01 (1.00,1.01) | 0.0004 | 55 (16) | 53 (18) | 1.00 (1.00,1.00) | 0.9970 |
| Charlson Index | mean (SD) | 1 (1) | 1 (1) | 0.98 (0.94,1.01) | 0.2034 | 2 (1) | 1 (2) | 0.72 (0.70,0.75) | <.0001 |

*Table 2: Summary of the number of admissions that resulted in an adverse event by whether or not they received Follow Up*

| **Variable** | **Variable** | **Not followed up** | **Followed up within 48hrs** | **Followed up within or outside 48hr** |
| --- | --- | --- | --- | --- |
| Readmission <= 28d | N~(n=44947) | 37146 (90%) | 5747 (92%) | 7801 (92%) |
|  | Y~(n=4774) | 4106 (10%) | 483 (7.8%) | 668 (7.9%) |
| 28d mortality | N~(n=49186) | 40792 (99%) | 6164 (99%) | 8394 (99%) |
|  | Y~(n=535) | 460 (1.1%) | 66 (1.1%) | 75 (0.9%) |
| Re-presentation to ED <=28d | N~(n=38441) | 31717 (77%) | 4940 (79%) | 6724 (79%) |
|  | Y~(n=11280) | 9535 (23%) | 1290 (21%) | 1745 (21%) |
| At least 1 adverse event | N~(n=37617) | 30977 (75%) | 4880 (78%) | 6640 (78%) |
|  | Y~(n=12104) | 10275 (25%) | 1350 (22%) | 1829 (22%) |

*Table 3. Crude (Unadjusted) Models for “Not followed up” compared to “Followed up within 48 hours”.*

|  | | **Readmission <= 28d** | | | | **28d mortality** | | | | **Representation to ED <=28d** | | | |
| --- | --- | --- | --- | --- | --- | --- | --- | --- | --- | --- | --- | --- | --- |
|  | | **N (%)** | | **GEE model** | | **N (%)** | | **GEE model** | | **N (%)** | | **GEE model** | |
| **Variable** | **Category** | **N (n=42893)** | **Y (n=4589)** | **OR (95% CI)** | **P-val** | **N (n=46956)** | **Y (n=526)** | **OR (95% CI)** | **P-val** | **N (n=36657)** | **Y (n=10825)** | **OR (95% CI)** | **P-val** |
| Followup | Not followed up | 37146 (90%) | 4106 ( 10%) | ref | 0.2937 | 40792 (99%) | 460 (1.1%) | ref | 0.5751 | 31717 (77%) | 9535 (23%) | ref | 0.2287 |
|  | Followed up within 48hrs | 5747 (92%) | 483 (7.8%) | 0.90 (0.73, 1.10) |  | 6164 (99%) | 66 (1.1%) | 0.93 (0.71, 1.21) |  | 4940 (79%) | 1290 (21%) | 0.95 (0.88, 1.03) |  |
| Care type | Centralised (SES HNE IS NBM SWS) | 17730 (90%) | 1928 (9.8%) | ref | 0.4172 | 19437 (99%) | 221 (1.1%) | ref | 0.8670 | 14666 (75%) | 4992 (25%) | ref | <0.0001 |
|  | Integrated (MNC WS NSYD) | 6687 (89%) | 861 (11%) | 1.17 (0.96, 1.43) |  | 7467 (99%) | 81 (1.1%) | 0.96 (0.73, 1.26) |  | 5586 (74%) | 1962 (26%) | 0.96 (0.87, 1.05) |  |
|  | Localised (SYD NNSW SNSW CC WNSW MUR) | 17918 (91%) | 1732 (8.8%) | 1.00 (0.86, 1.17) |  | 19432 (99%) | 218 (1.1%) | 0.98 (0.79, 1.20) |  | 15938 (81%) | 3712 (19%) | 0.75 (0.70, 0.81) |  |
|  | Mixed LHD (FW) | 535 (89%) | 67 (11%) | 1.27 (0.83, 1.95) |  | 596 (99%) | 6 (1.0%) | 0.66 (0.24, 1.79) |  | 448 (74%) | 154 (26%) | 0.93 (0.71, 1.22) |  |
|  | Other | 23 (96%) | 1 (4.2%) | 0.97 (0.07, 13.44) |  | 24 (100%) |  |  |  | 19 (79%) | 5 (21%) | 0.91 (0.28, 2.91) |  |
| Year | 2009 | 6234 (90%) | 698 (10%) | ref | 0.0429 | 6863 (99%) | 69 (1.0%) | ref | 0.0019 | 5544 (80%) | 1388 (20%) | ref | <0.0001 |
|  | 2010 | 6192 (91%) | 576 (8.5%) | 0.86 (0.64, 1.15) |  | 6698 (99%) | 70 (1.0%) | 1.09 (0.75, 1.57) |  | 5358 (79%) | 1410 (21%) | 1.09 (0.97, 1.22) |  |
|  | 2011 | 6564 (90%) | 715 (9.8%) | 1.14 (0.87, 1.50) |  | 7200 (99%) | 79 (1.1%) | 1.14 (0.78, 1.65) |  | 5624 (77%) | 1655 (23%) | 1.20 (1.07, 1.34) |  |
|  | 2012 | 8274 (90%) | 912 (9.9%) | 1.16 (0.89, 1.50) |  | 9064 (99%) | 122 (1.3%) | 1.44 (1.03, 2.02) |  | 6921 (75%) | 2265 (25%) | 1.29 (1.16, 1.45) |  |
|  | 2013 | 10315 (90%) | 1176 (10%) | 1.27 (0.99, 1.63) |  | 11343 (99%) | 148 (1.3%) | 1.43 (1.04, 1.97) |  | 8639 (75%) | 2852 (25%) | 1.35 (1.21, 1.50) |  |
|  | 2014 | 5314 (91%) | 512 (8.8%) | 1.02 (0.77, 1.37) |  | 5788 (99%) | 38 (0.7%) | 0.76 (0.51, 1.14) |  | 4571 (78%) | 1255 (22%) | 1.17 (1.04, 1.32) |  |
| Gender | Male | 19468 (90%) | 2145 (9.9%) | ref | 0.0091 | 21341 (99%) | 272 (1.3%) | ref | 0.0065 | 16484 (76%) | 5129 (24%) | ref | 0.0010 |
|  | Female | 23229 (90%) | 2440 (9.5%) | 0.88 (0.80, 0.97) |  | 25415 (99%) | 254 (1.0%) | 0.77 (0.64, 0.93) |  | 19988 (78%) | 5681 (22%) | 0.90 (0.85, 0.96) |  |
| Marital status | Married/defacto | 16086 (91%) | 1521 (8.6%) | ref | 0.0004 | 17405 (99%) | 202 (1.1%) | ref | <0.0001 | 13978 (79%) | 3629 (21%) | ref | <0.0001 |
|  | Single | 13977 (89%) | 1659 (11%) | 1.06 (0.91, 1.25) |  | 15519 (99%) | 117 (0.7%) | 0.64 (0.50, 0.83) |  | 11645 (74%) | 3991 (26%) | 1.16 (1.07, 1.24) |  |
|  | Widowed | 5143 (88%) | 679 (12%) | 1.53 (1.26, 1.86) |  | 5699 (98%) | 123 (2.1%) | 1.87 (1.47, 2.39) |  | 4389 (75%) | 1433 (25%) | 1.28 (1.15, 1.42) |  |
|  | Divorced/separated | 5784 (90%) | 628 (9.8%) | 1.25 (1.01, 1.55) |  | 6343 (99%) | 69 (1.1%) | 0.97 (0.72, 1.31) |  | 4959 (77%) | 1453 (23%) | 1.21 (1.09, 1.33) |  |
|  | Not known | 428 (92%) | 36 (7.8%) | 0.94 (0.37, 2.44) |  | 459 (99%) | 5 (1.1%) | 0.98 (0.40, 2.42) |  | 377 (81%) | 87 (19%) | 1.07 (0.80, 1.44) |  |
| IRSD quintile | 1st quintile -least disadvantaged | 7090 (89%) | 842 (11%) | ref | 0.4293 | 7835 (99%) | 97 (1.2%) | ref | 0.8150 | 5863 (74%) | 2069 (26%) | ref | <0.0001 |
|  | 2nd quintile | 8050 (90%) | 895 (10%) | 1.04 (0.82, 1.32) |  | 8841 (99%) | 104 (1.2%) | 0.98 (0.72, 1.32) |  | 6795 (76%) | 2150 (24%) | 0.92 (0.83, 1.02) |  |
|  | 3rd quintile | 9290 (90%) | 996 (9.7%) | 1.09 (0.87, 1.36) |  | 10168 (99%) | 118 (1.1%) | 0.93 (0.69, 1.25) |  | 7808 (76%) | 2478 (24%) | 0.98 (0.89, 1.08) |  |
|  | 4th quintile | 8149 (91%) | 837 (9.3%) | 0.90 (0.72, 1.12) |  | 8885 (99%) | 101 (1.1%) | 0.91 (0.67, 1.24) |  | 7136 (79%) | 1850 (21%) | 0.80 (0.72, 0.88) |  |
|  | 5th quintile - most disadvantaged | 8813 (90%) | 952 (9.7%) | 0.97 (0.75, 1.24) |  | 9669 (99%) | 96 (1.0%) | 0.84 (0.62, 1.14) |  | 7730 (79%) | 2035 (21%) | 0.78 (0.70, 0.87) |  |
| Participation in the CDMP | Did not participate | 41954 (91%) | 4247 (9.2%) | ref | 0.0168 | 45723 (99%) | 478 (1.0%) | ref | <0.0001 | 35938 (78%) | 10263 (22%) | ref | <0.0001 |
|  | Participated | 939 (73%) | 342 (27%) | 1.87 (1.12, 3.11) |  | 1233 (96%) | 48 (3.7%) | 3.96 (2.90, 5.41) |  | 719 (56%) | 562 (44%) | 1.77 (1.45, 2.16) |  |
| Participation in the Healthways component | Did not participate | 42868 (90%) | 4581 (9.7%) | ref | 0.2546 | 46923 (99%) | 526 (1.1%) | Cannot be added into model due to zero counts | | 36638 (77%) | 10811 (23%) | ref | 0.1825 |
|  | Participated | 25 (76%) | 8 (24%) | 1.98 (0.61, 6.46) |  | 33 (100%) |  |  |  | 19 (58%) | 14 (42%) | 1.62 (0.80, 3.30) |  |
| Length of stay | 1 day or less | 16226 (92%) | 1464 (8.3%) | ref | 0.2326 | 17595 (99%) | 95 (0.5%) | ref | <0.0001 | 13984 (79%) | 3706 (21%) | ref | <0.0001 |
|  | More than 1 day | 25220 (89%) | 3062 (11%) | 1.09 (0.94, 1.27) |  | 27861 (99%) | 421 (1.5%) | 2.70 (2.14, 3.41) |  | 21389 (76%) | 6893 (24%) | 1.15 (1.08, 1.22) |  |
| No of previous admissions | None | 17043 (96%) | 751 (4.2%) | ref | 0.0049 | 17678 (99%) | 116 (0.7%) | ref | <0.0001 | 14945 (84%) | 2849 (16%) | ref | <0.0001 |
|  | 1 or more | 25850 (87%) | 3838 (13%) | 1.21 (1.06, 1.37) |  | 29278 (99%) | 410 (1.4%) | 2.30 (1.87, 2.83) |  | 21712 (73%) | 7976 (27%) | 1.28 (1.21, 1.35) |  |
| No of additional diagnoses | Less than 2 | 12804 (93%) | 925 (6.7%) | ref | 0.0325 | 13655 (99%) | 74 (0.5%) | ref | <0.0001 | 11307 (82%) | 2422 (18%) | ref | <0.0001 |
|  | 2 or more | 30089 (89%) | 3664 (11%) | 1.19 (1.01, 1.40) |  | 33301 (99%) | 452 (1.3%) | 2.53 (1.93, 3.33) |  | 25350 (75%) | 8403 (25%) | 1.24 (1.16, 1.32) |  |
| Age | mean (SD) | 54 (17) | 56 (17) | 1.02 (1.01, 1.02) | <0.0001 | 54 (17) | 65 (13) | 1.05 (1.04, 1.05) | <0.0001 | 55 (17) | 54 (17) | ref | 0.0803 |
| Charlson Index | mean (SD) | 2 (2) | 2 (1) | 1.13 (1.09, 1.18) | <0.0001 | 2 (1) | 3 (2) | 1.51 (1.45, 1.57) | <0.0001 | 2 (1) | 2 (2) | 1.11 (1.09, 1.13) | <0.0001 |
